# Characterization of the CpG island methylator phenotype subclass in papillary thyroid carcinoma

**DOI:** 10.3389/fendo.2022.1008301

**Published:** 2022-10-24

**Authors:** Pengfei Gu, Yu Zeng, Weike Ma, Wei Zhang, Yu Liu, Fengli Guo, Xianhui Ruan, Jiadong Chi, Xiangqian Zheng, Ming Gao

**Affiliations:** ^1^ Department of Thyroid and Neck Cancer, Tianjin Medical University Cancer Institute and Hospital, National Clinical Research Center for Cancer, Key Laboratory of Cancer Prevention and Therapy, Tianjin’s Clinical Research Center for Cancer, Tianjin, China; ^2^ Department of Thyroid and Breast Surgery, Tianjin Union Medical Center, Tianjin, China; ^3^ Tianjin Key Laboratory of General Surgery in Construction, Tianjin Union Medical Center, Tianjin, China

**Keywords:** CpG island methylator phenotype, papillary thyroid carcinoma, immune-depletion, prognosis, epigenetic

## Abstract

CpG island methylator phenotype (CIMP), characterized by the concurrent and widespread hypermethylation of a cluster of CpGs, has been reported to play an important role in carcinogenesis. Limited studies have explored the role of CIMP in papillary thyroid carcinomas (PTCs). Here, in genome-wide DNA methylation analysis of 350 primary PTCs from the Cancer Genome Atlas database that were assessed using the Illumina HumanMethylation450K platform, our study helps to identify two subtypes displayed markedly distinct DNA methylation levels, termed CIMP (high levels of DNA methylation) and nCIMP subgroup (low levels of DNA methylation). Interestingly, PTCs with CIMP tend to have a higher degree of malignancy, since this subtype was tightly associated with older age, advanced pathological stage, and lymph node metastasis (all *P* < 0.05). Differential methylation analysis showed a broad methylation gain in CIMP and subsequent generalized gene set testing analysis based on the significantly methylated probes in CIMP showed remarkable enrichment in epithelial mesenchymal transition and angiogenesis hallmark pathways, confirming that the CIMP phenotype may promote the tumor progression from another perspective. Analysis of tumor microenvironment showed that CIMP PTCs are in an immune-depletion status, which may affect the effectiveness of immunotherapy. Genetically, the significantly higher tumor mutation burden and copy number alteration both at the genome and focal level confirmed the genomic heterogeneity and chromosomal instability of CIMP. tumor Corresponding to the above findings, PTC patients with CIMP showed remarkable poor clinical outcome as compared to nCIMP regarding overall survival and progression-free survival. More importantly, CIMP was associated with worse survival independent of known prognostic factors.

## Introduction

Papillary thyroid carcinoma (PTC) is the most common pathologic type of thyroid carcinoma (TC), accounting for about 85% of all cases. In recent years, the incidence and recurrence rate of PTC is gradually increasing throughout the world (1, 2). Although PTC has always been regarded as an indolent malignancy, with a 10-year relative survival rate of 98% in cases with localized disease (3), a subset of them show poor outcomes (4). A great number of patients with PTC develop locoregional recurrences or even radioiodine-resistant distant metastases (5, 6). Furthermore, PTC occasionally give rise to less differentiated and more aggressive TCs (5). It is indeed a challenge for clinicians to provide the most effective but least aggressive treatment (7). Considering the high and still rising incidence of PTC whose adverse outcomes may sometimes be ignored, it is necessary to perform more precise therapies, or to search for novel prognostic markers to perfect the risk stratification process.

DNA methylation is the most well-known epigenetic modification, occurring from the addition of a methyl group to the 5’-position of the cytosine of cytosine-guanine dinucleotides. Previous studies have outlined the genome-wide landscape of cancer-specific DNA methylation changes, which is characteristic of global hypomethylation and a regional hypermethylation in CpG islands (CGIs) (8–10). The former is considered to favour chromosomal instability and inappropriate activation of oncogenes, the latter may lead to the - silencing of tumor-suppressor genes (8, 11). Changes in DNA methylation in cancer have been regarded as promising targets for the development of powerful diagnostic, prognostic, and predictive biomarkers (12, 13). Although DNA methylation in PTC has been intensively studied and several markers have been described, most of the research are mainly based on the analysis of candidate genes (14, 15), only a limited number of genome-wide methylation studies have been reported in PTC (16, 17).

The CpG island methylator phenotype (CIMP) was first discovered and validated in colorectal cancer, as cancer-specific CGIs hypermethylation of a subset of genes in a subset of tumor (18). Occurrence of CIMP is associated with a range of genetic and environmental factors, although the molecular causes are not well-understood (19). So far, the CIMP phenotype has been identified in many kinds of tumors, including glioma (20), renal cell carcinoma (21), gastric cancer (22), Pancreatic Cancer (23), and so on. Subtypes with different CIMP patterns showed distinct clinical-pathological, genomic, and immune-related characteristics (24, 25). However, whether CIMP presents a biological subtype and whether this phenotype is relevant to tumorigenesis and progression of PTC has not been reported.

In this study, we identified and validated two distinct methylation subgroups of PTC patients, termed the CIMP subgroup (High levels of DNA methylation) and nCIMP subgroup (Low levels of DNA methylation), with data downloaded from the Cancer Genome Atlas (TCGA) and further investigated their impact on the patient prognosis, immune profiles, epigenetic alterations and response to immune therapy or chemotherapeutic drugs. As identification of clinically relevant cancer subtypes based on DNA methylation patterns, CIMP might serve as a tool for precisely risk stratification and help to make medication guide for patients of different subtypes.

## Methods

### Multi-omics data sets of TCGA-THCA

DNA methylation profile quantified by Illumina HumanMethylation 450K-array platform was downloaded from the UCSC Xena (https://xenabrowser.net/) under the project of TCGA-THCA (17), including 502 primary TC and 54 adjacent normal samples. Within these cases, we identified 353 PTC cases according to the record of histology. For transcriptome profile, we downloaded the gene expression data for 497 primary TC and 56 adjacent normal samples quantified by the number of fragments per kilobase million (FPKM); FPKM values were subsequently converted into transcripts per kilobase million, which showed more similarity to the numbers obtained from microarray analysis and improved comparability between samples (26). In these 497 primary tumors with available transcriptome profile, a total of 350 PTCs were identified which also had matched DNA methylation profile. In addition, 48 adjacent normal samples were shared between DNA methylation and gene expression profiles. Additionally, copy number segment data were collected from FireBrowse (http://firebrowse.org/). Somatic mutations, clinicopathological features, overall survival (OS) and progression-free survival (PFS) rate data were downloaded from cBioPortal (https://www.cbioportal.org/).

### External transcriptome datasets

According to the literature (27), we collected a total of five PTC datasets from Gene Expression Omnibus that were sequenced by Affymetrix genechip, including GSE33630, GSE60542, GSE3467, GSE3678 and GSE27155. Considering the stability and robustness of the validation analysis, those datasets with tumor sample size greater than 30 were kept, including GSE33630 (n = 49) (28), GSE60542 (n = 33) (29), and GSE27155 (n = 51) (30, 31). For microarray data, the median value was considered if the gene symbol was annotated with multiple probe IDs.

### Pre-processing of DNA methylation profile

For DNA methylation profile, we used the R package “*ChAMP*” to performed comprehensive filtering procedures and the following filtering criteria were adopted: removal of probes with detection *P* value > 0.01 and probes with < 3 beads in at least 5% of samples per probe, all non-CpG probes, all single nucleotide polymorphism-related probes, all multi-hit probes, and probes locating in chromosome X and Y (32, 33).

### Unsupervised clustering of DNA methylation profile

Probes that were unmethylated in the 48 normal samples (mean β-value < 0.3) and that had a standard deviation (SD) of greater than 0.15 in the tumor samples were chosen for the clustering. In addition to β-values, we used M-values in this study (M-value = log2(β/1 β) because of the stronger signals for quantifying methylation levels (34). Unsupervised hierarchical clustering with Ward’s method and Euclidean distance measurement was used to cluster the 350 primary tumor samples based on methylation M-values, and the clustering dendrogram was cut at k = 2 to yield two clusters.

### Calculation of microenvironment cell abundance and pathway enrichment

We used the R package “*ESTIMATE*” (35) to estimate the presence of infiltrating immune/stromal cells in tumor tissue. Furthermore, the score of DNA methylation of tumor-infiltrating lymphocyte (MeTIL) in the TCGA-PTC cohort was calculated individually according to the protocols outlined in the literature (36). We also quantified the absolute abundance of eight immune cell populations and two stromal cell populations in heterogeneous tissues by the R package “*MCPcounter*” (37).

### Differential analysis and functional enrichment

The differential methylation probes were obtained through the standard process of ChAMP based on the following criteria. We determined probe as the probe that significantly gained methylation if its corresponding mean β value was greater than 0.4 in the specific subtype but less than 0.3 in the reference group with *P* < 0.05 and false discovery rate (FDR) < 0.25; vice versa for probes that significantly lost methylation. Functional enrichment analysis through generalized gene set testing (GGST) was performed for CpG level of DNA methylation by using the R package “*missMethyl*” with the Hallmark gene set background retrieved from Molecular Signatures Database (38, 39). Differential expression analyses were conducted using the R package “*limma*” (40). For gene set enrichment analysis (GSEA) based on gene expression data, pre-ranked gene list was prepared according to the descending ordered log2FoldChange value derived from differential expression analysis; we then harnessed R package “*clusterProfiler*” to determine functional enrichment based on Hallmark pathway (41). Functional enrichment analysis based on gene list was performed by Enrichr (https://maayanlab.cloud/Enrichr/) (42).

### Characterization of cancer subtype

Cancer subtypes we identified were basically characterised by the R package “*MOVICS*”, including mutational frequency, fraction of copy number-altered genome, and clinical characteristics using all parameters by default (43). Additionally, we analysed the mutation landscape by the R package “*maftools*” using the potential driver mutation according to the literature (44, 45). Recurrent focal somatic copy number alterations(CNAs) were detected and localized by GISTIC2.0 through GenePattern (https://www.genepattern.org/) with all parameters by default (46). Arm-level chromosome CNA status were retrieved from the previous literature (47).

### Integrative analysis of promoter DNA methylation and transcriptome expression

To investigate the crosstalk between DNA methylation and transcriptome expression, we performed integrative analysis combining DNA methylation and gene expression by using the R package “*ELMER*” (48). First, we identified probes that located in promoters from the annotation of Infinium HumanMethylation450K BeadChip. Secondly, we identified putative genes that were significantly downregulated due to the hypermethylation of promoter probes. Thirdly, the closest 20 upstream and downstream genes were collected for each probe, and for each candidate probe-gene pair, the Mann-Whitney U test was harnessed to test the null hypothesis that overall gene expression in the specific group was less than or equal than that in the reference group.

### Prediction of the benefit from immune checkpoint blockade therapy and drug sensitivity

The MD Anderson melanoma cohort that received anti CTLA-4 or anti-PD-1 therapy was considered for the prediction of immunotherapy response (49). In addition, based on the drug sensitivity and phenotype data from GDSC 2016 (https://www.cancerrxgene.org/), the R package “*pRRophetic*” was employed to predict the chemotherapeutic sensitivity for each PTC sample using the expression profiles of 727 human cancer cell lines as the training cohort; the 50% inhibiting concentration (IC_50_) (lower IC_50_ indicates increased sensitivity to treatment) of each sample treated with a specific chemotherapeutic agent was estimated by ridge regression, and 10-fold cross-validation was used to measure the prediction accuracy (50).

### Statistical analyses

All statistical analyses including Fisher’s exact test for categorical data, a two-sample Mann-Whitney U test for continuous data, a log-rank test Kaplan-Meier curve, and hazard ratio with 95% confidence interval for Cox proportional hazards regression, and unsupervised hierarchical clustering with Ward’s method and Euclidean distance measurement based on methylation M-values (M-value = log2(β/1 β), were performed by R4.0.2. A two-sided P<0.05 was considered statistically significant in all unadjusted methods of comparison.

## Results

### Identification of a CpG island methylator phenotype in PTC associated with patient outcome

**Figure 1:**
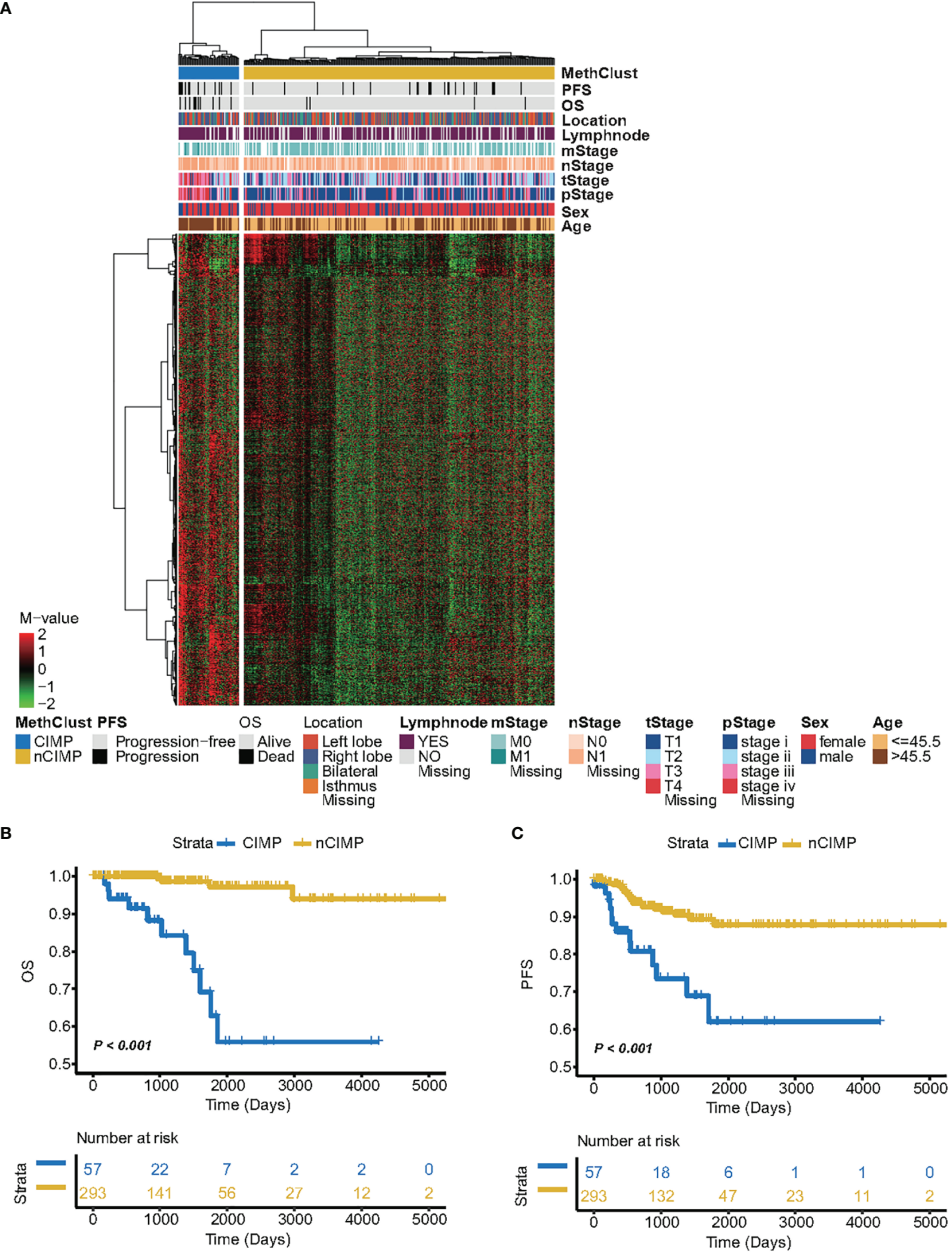
Identification of CIMP and its association with clinical outcome. **(A)** Heatmap showing the DNA methylation pattern between CIMP (high-methylation) and nCIMP(low-methylation) using DNA methylation M-values. Kaplan-Meier curves between two epigenetic phenotypes regarding **(B)** OS (Overall survival) and **(C)** PFS (Progression free survival).

We analysed DNA methylation profile of 350 primary PTCs from TCGA database accessed by the Illumina HumanMethylation450K platform. After a comprehensive filtering of ChAMP procedure, a total of 340,864 probes remained for the analysis. We then excluded totally 191,341 probes with average β-value > 0.3 in 48 normal thyroid samples. After that, the probes with high variability (SD > 0.15) were selected, leading to a total of 6,541 probes used for the clustering analysis. Consequently, unsupervised hierarchical clustering based on these probes revealed two subtypes, one of which displayed markedly high DNA methylation levels and thus be labelled as having a CIMP phenotype (n = 57, 16.3%). Another subclass exhibited low methylation levels and was therefore termed nCIMP (n = 293, 83.7%) (**Figure 1A**).

Next, we aimed to assess whether PTCs belong to different CIMP phenotypes exhibited distinct clinicopathologic features. Interestingly, the results showed that CIMP tumors were tightly associated with several adverse prognostic factors, such as older age (*P* < 0.001), advanced T stage (*P* < 0.001), severer pathological stage (*P* < 0.001), and lymph node metastasis (*P* = 0.039) (**Supplementary Table S1**). The distribution between the CIMP/nCIMP phenotype and clinicopathological features were shown in **Supplementary Figure S1**. Furthermore, Kaplan-Meier analysis was used to investigate the association between the above two phenotypes and patients’ clinical outcome. Surprisingly, we found that PTC patients with CIMP showed significantly poor clinical outcome as compared to nCIMP regarding OS (*P* < 0.001; **Figure 1B**) and PFS (*P* < 0.001; **Figure 1C**), which suggested an interplay between DNA methylation and known prognostic features in PTC.

### Broad methylation gain in CIMP

After that, we performed differential methylation analysis between CIMP and nCIMP and identified differentially methylated probes for each phenotype. Specifically, a total of 929 probes were identified as significantly methylated in CIMP compared to only 13 probes in nCIMP (All *P* < 0.05, **Supplementary Table S2**), indicating a significant increase of DNA methylation in CIMP. Simultaneously, GGST analysis based on these 929 probes showed significantly enrichment in epithelial mesenchymal transition and angiogenesis Hallmark pathways (both *P* < 0.05; **Supplementary Table S3**, **Supplementary Figure S2**).

### Differential immune profiles between two epigenetic phenotypes

**Figure 2:**
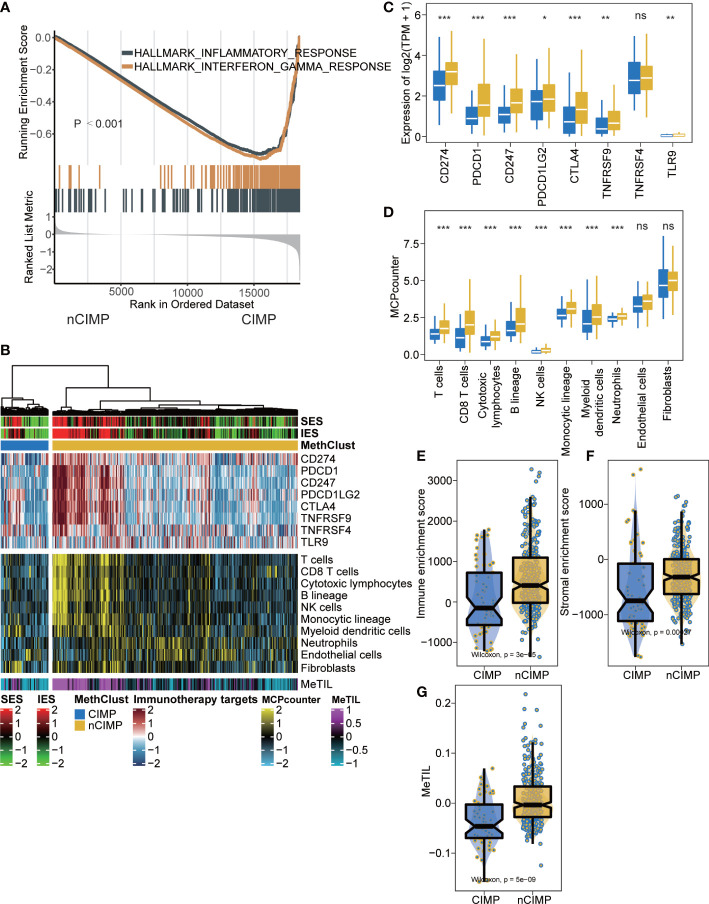
Tumor microenvironment landscape of CIMP. **(A)** GSEA plot showing inactivation of inflammatory response and interferon-γ. **(B)** Heatmap showing the immune profile in the TCGA-PTC cohort, with the top panel showing the expression of genes involved in immune checkpoint targets, the middle panel showing the enrichment level of 10 microenvironment cell types, and the bottom panel showing the DNA methylation of tumor-infiltrating lymphocytes (MeTILs). The immune enrichment score and stromal enrichment score were annotated at the top of the heatmap. **(C)** Boxplot showing the distribution of expression of immune checkpoint target genes between two epigenetic phenotypes. **(D)** Boxplot showing the distribution of enrichment score of 10 microenvironment cell types between two epigenetic phenotypes. Distribution of immune enrichment scores, stromal enrichment scores and MeTIL scores between two epigenetic phenotypes were shown in **(E–G)**, respectively (ns stands for no significance, ^*^p < 0.05, ^**^p < 0.01, ^***^p < 0.001).

To investigate the transcriptional changes between the two epigenetic phenotypes, differential expression analysis and GSEA were conducted. The results showed significant inactivation of inflammatory response and interferon-γ Hallmark pathways in CIMP as compared to nCIMP (both FDR < 0.001; **Figure 2A**, **Supplementary Table 4**).

Since cancer immunity plays a critical role in tumor progression, we suspected that the tumor microenvironment (TME) of the CIMP may be different from that of the nCIMP subtype. Thus, we investigated the specific TME cell infiltration status of samples from TCGA; the infiltration levels of eight immune and two stromal cell populations were quantified and the expression of immune checkpoints in PTC samples were investigated (**Figure 2B**).

As expected, the expression of genes representing potential targets of immunotherapy, including PDCD1 (PD1), CD247 (CD3), CD274 (PDL1), PDCD1LG2 (PDL2), CTLA4 (CD152), TNFRSF9 (CD137) and TLR9 (all, P < 0.05), was in CIMP significantly lower than that of nCIMP (**Figure 2C**). The analysis of TME suggested that CIMP was dramatically immune-depleted because all eight quantified level of immune cells were significantly lower than that of nCIMP (all, *P* < 0.05; **Figure 2D**). Using the ESTIMATE algorithm, we found that CIMP presented with overall lower enrichment level regarding immune and stromal cells (both, *P* < 0.001; **Figures 2E, F**). Additionally, the MeTIL score of CIMP in TCGA cohort was significantly lower than that of nCIMP, indicating a lower proportion of tumor-infiltrating leukocytes (*P* < 0.001, **Figure 2G**).

### Epigenetically silence of immune-related pathways

**Figure 3:**
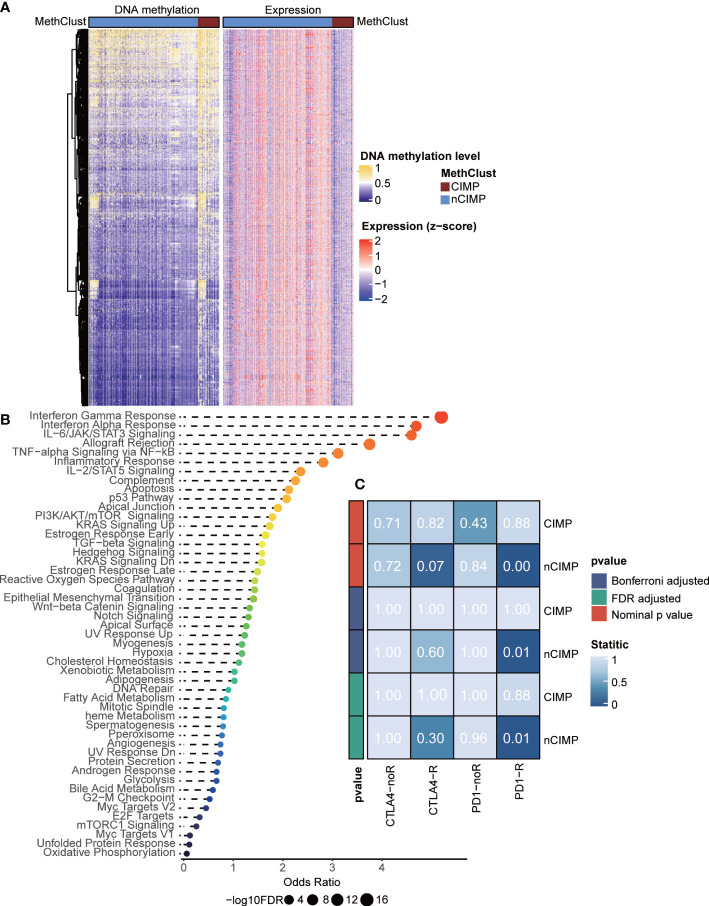
Integrated analysis of DNA methylation and transcriptome gene expression, and prediction of sensitivity of methylation phenotypes to immune checkpoint therapy. **(A)** Heatmap showing the association between DNA methylation and gene expression, presenting with an epigenetic silencing pattern. **(B)** Dotplot showing the Enrichr functional enrichment of 1,467 adjacent genes. **(C)** Heatmap of subclass analysis result showing the differences of sensitivity to several immune checkpoint inhibitors between the CIMP and nCIMP groups.

Given the immune-depleted TME in CIMP, we then decided to investigate if such transcriptional change could mirror to epigenetic DNA methylation. In this context, we performed integrative analysis combining both gene expression and DNA methylation profiles using ELMER pipeline. Due to the well-known epigenetic effect of promoter methylation in silencing corresponding gene expression, we extracted promoter probes from the Illumina HumanMethylation 450K-array platform, and performed differential methylation analysis in probe level. Probes with difference of β-value greater than 0.1 (FDR < 0.05) between CIMP and nCIMP were identified, ending up with a total of 2,404 promoter probes (**Supplementary Table S5**). Next, ELMER was employed to search for 20 adjacent genes corresponding to these probes (**Supplementary Table S6**), and further predicted promoter-gene linkages using associations between DNA methylation at promoter CpG sites and expression of 20 adjacent genes of the CpG sites (**Figure 3A**); such analysis identified to a total of 3,272 gene pairs, corresponding to 1,467 different genes (**Supplementary Table S7**). To understand the biologic relevance of these genes that were epigenetically silenced, Enrichr was employed and found that these genes were significantly enriched in inflammatory response and interferon-γ Hallmark pathways (both, FDR < 0.001; **Figure 3B**, **Supplementary Table S8**).

### CIMP may not benefit from immunotherapy

Considering the dramatical diversity of TME, we next investigated whether there is a difference among the two phenotypes in the likelihood of responding to immune checkpoint blockade. To this end, we performed subclass mapping in TCGA cohort and revealed that the nCIMP showed high transcriptional similarity with a subgroup of melanoma patients who responded to anti-PD1 blockade (adjusted *P* < 0.05; **Figure 3C**), which indicated that patients in the nCIMP subgroup can profit more from anti-PD-1 treatment.

### Validation of TME in external PTC cohorts

**Figure 4:**
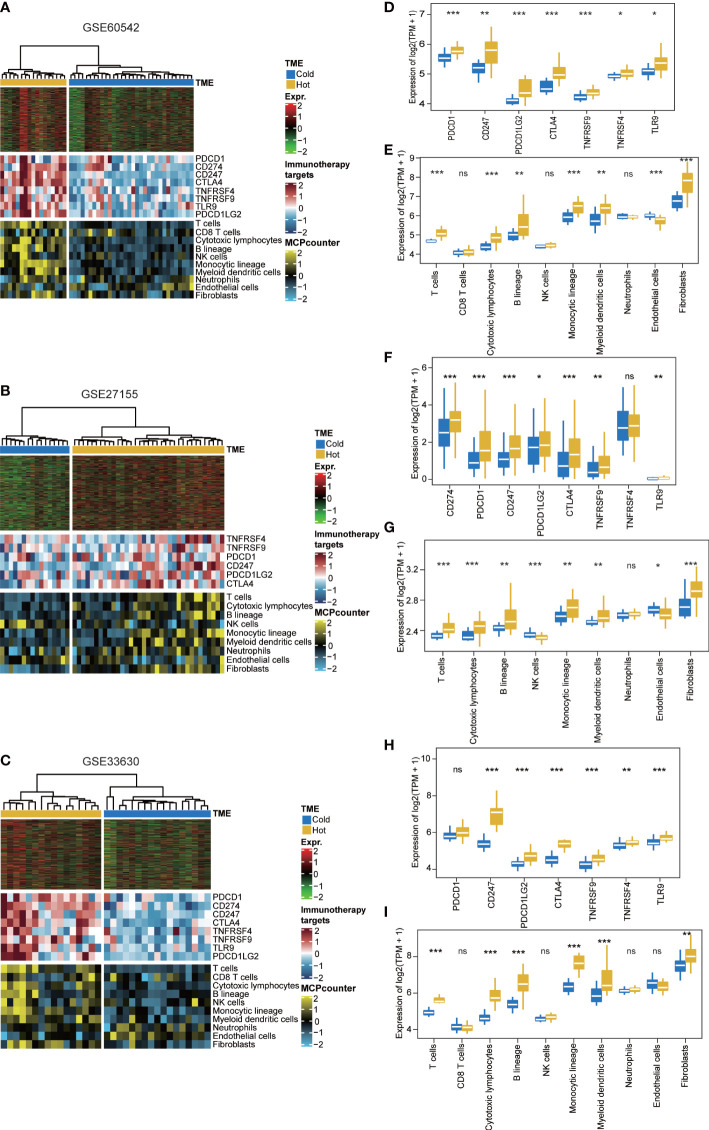
Validation of TME using supervised clustering based on 1,467 epigenetically silenced genes. The heat map showed the expression of these genes (top panel), genes representing immune checkpoint targets (middle panel), and immune and stromal cells (bottom panel) in three validation cohorts, including **(A)** GSE60542, **(B)** GSE27155, and **(C)** GSE33630. **(D, F, H)** Expression level (normalized transcripts per million) of different immune checkpoint genes in different methylation phenotypes of three validation cohorts. **(E, G, I)** The boxplot showed the accumulation of immune and stromal cell populations distinguished by different methylation phenotypes in the three validation cohorts. The difference was verified statistically through the Kruskal–Wallis test, and the p-values are noted with asterisks at the top of each boxplot (ns stands for no significance, ^*^p < 0.05, ^**^p < 0.01, ^***^p < 0.001).

Due to the lack of public PTC cohorts with available DNA methylation data, we retrieved three PTC cohorts with available RNA-seq gene expression profile to test the reproducibility of the immune-depleted phenotype using the 1,467 genes that may be silenced by promoter hypermethylation. To this end, we performed supervised hierarchical clustering and revealed two subtypes in each of the external cohort. Of note, all three cohorts existed a “cold” TME phenotype that showed immune-depleted landscape (**Figures 4A-I**).

### Genomic heterogeneity and chromosomal instability of CIMP

**Figure 5:**
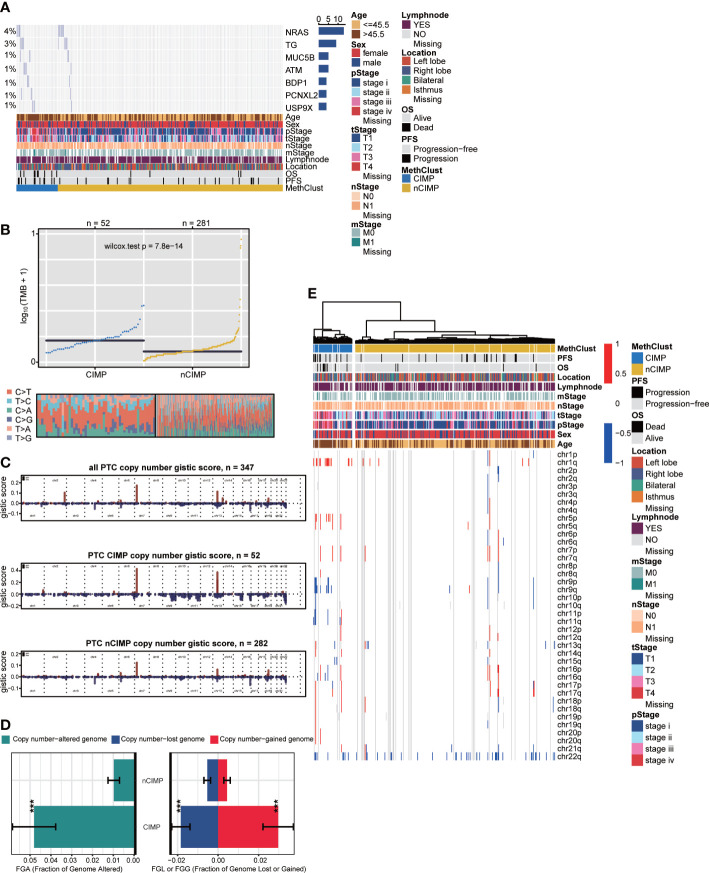
Genomic heterogeneity and chromosomal instability of CIMP. **(A)** OncoPrint showing the distribution of genes that were differentially mutated between two epigenetic phenotypes. **(B)** Distribution of TMB and TiTv (transition to transversion) between two epigenetic phenotypes. **(C)** Barplot showing the distribution of FGA and fraction genome gain/loss (FGG/FGL). Bar charts are presented as the mean ± standard error of the mean. **(D)** Comparison of focal-level CNA across entire cohort and in different epigenetic phenotypes, respectively. **(E)** Heatmap showing the distribution of arm-level CNA between two epigenetic phenotypes (^***^p < 0.001).

To investigate the genomic heterogeneity of PTC, we analysed the mutational landscape and identified seven genes across the entire cohort that showed differential mutational frequency between two phenotypes (*P* < 0.05) with overall mutational rate greater than 1%. These genes included *NRAS*, *TG*, *MUC5B*, *ATM*, *BDP1*, *PCNXL2* and *USP9X* (**Figure 5A**, **Supplementary Table S9**). Among genes that were previously identified as driver mutations for TC (44), we found that CIMP contained significantly more NRAS mutations comparing to nCIMP. Additionally, CIMP had significantly higher tumor mutation burden (TMB, *P* < 0.001) than that of nCIMP (**Figure 5B**).

We then estimated the chromosomal instability by calculating the fraction genome alteration scores and found that nCIMP had better chromosomal stability than CIMP with significantly lower copy number loss or gain (both, *P* < 0.001; **Figure 5C**). Consistently, the landscape of focal CNA demonstrated highly instability of chromosome in CIMP against nCIMP (**Figure 5D**). Specifically, CIMP had more gain of chr1q, 5p, and loss of chr 9q, and 11q (all, FDR < 0.001; **Figure 5E**, **Supplementary Table S10**).

### Independent prognostic value of CIMP

**Figure 6:**
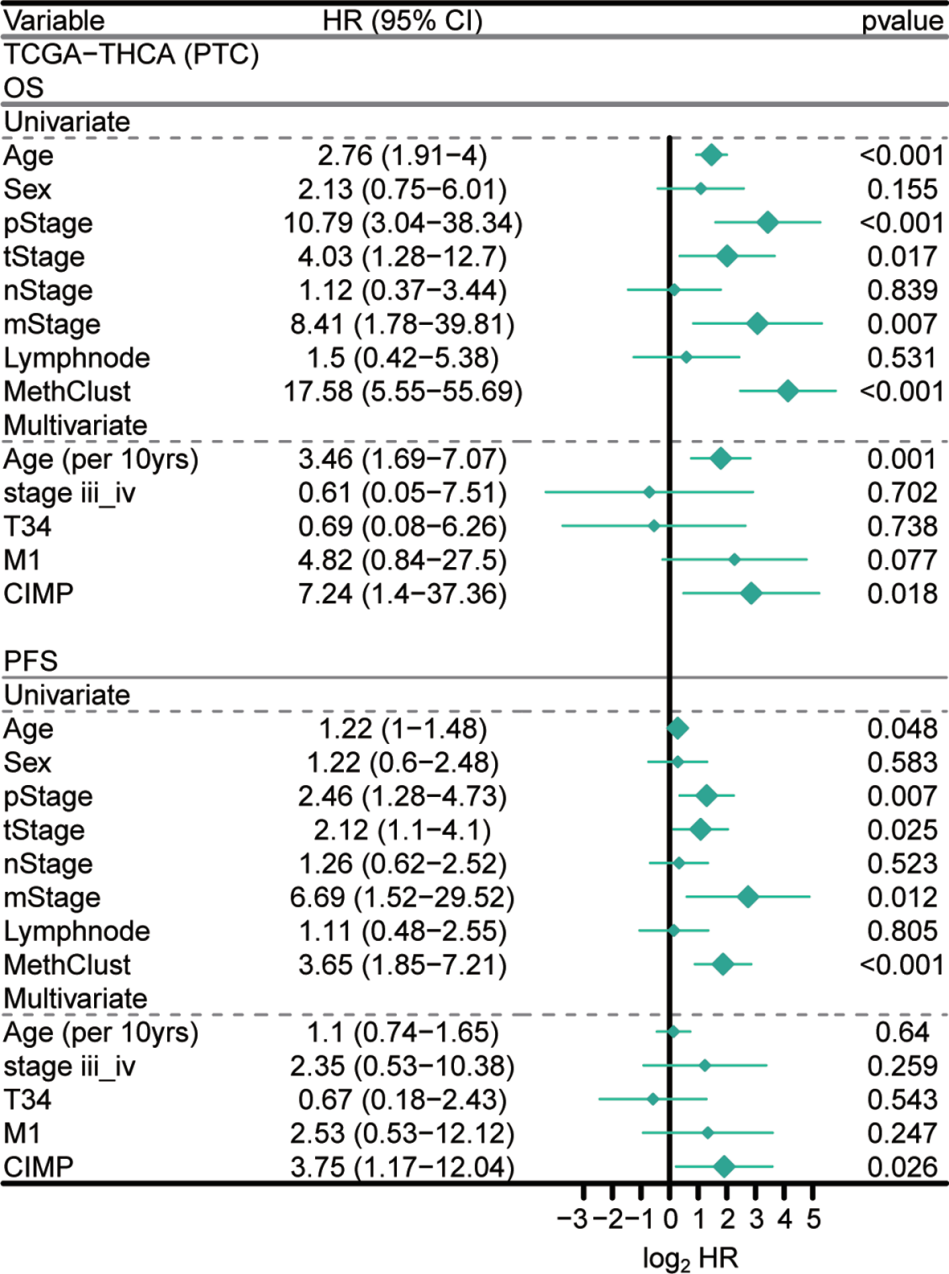
Independent prognostic value of CIMP. Forest plot showing the hazard ratio (95% CI) in univariate and multivariate Cox regressions with the corresponding *P* values.

We then surveyed that whether CIMP was an independent prognostic factor in PTC from TCGA cohort. In this manner, univariate Cox regression model was first conducted to filter out prognostic clinical characterizations concerning OS and PFS; multivariate Cox regression was subsequently performed based on those prognosis-relevant features. Using such strategy, we found that age, T stage, M stage, pathological stage and CIMP were prognostic using univariate analysis, and only CIMP remained the independent prognostic factor after adjusting these clinical prognostic features with respect to OS (*P* < 0.001) and PFS (*P* = 0.018) (**Figure 6**).

### Potential therapeutic strategy for CIMP

**Figure 7:**
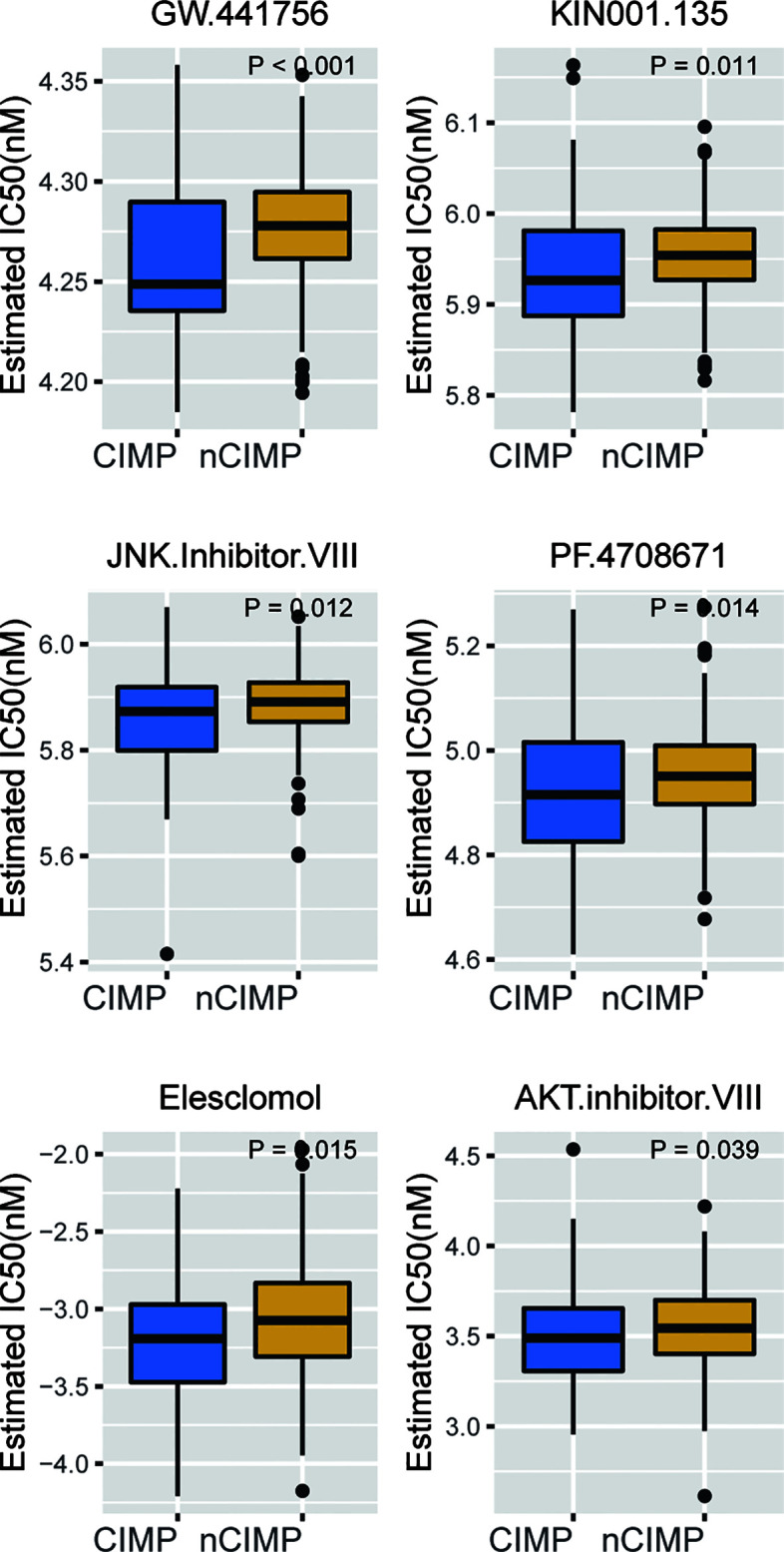
Identification of potential therapeutic drugs for CIMP. Boxplot showing the distribution of estimated IC50 between two epigenetic phenotypes based on GDSC database.

Considering the significantly poor clinical outcome of CIMP in PTC, we decided to infer potential anti-PTC drugs that were associated with CIMP using an *in-sillico* drug screening approach. To this end, we constructed ridge regression model between cell lines and corresponding drug sensitivity and applied the predictive model to each of the PTC cases (**Supplementary Table S11**). A lower estimated IC50 value was obtained in the CIMP group compared to the nCIMP group. This result suggests that in PTC patients, the higher the degree of methylation of CPGs, then the more sensitive the patients may be to certain therapeutic agents and their therapeutic outcome is better, including GW.441756, KIN001.135, JNK Inhibitor VIII, PF.4708671, Elesclomol, and AKT Inhibitor VII (all, *P* < 0.05; **Figure 7**)

## Discussion

Alterations in DNA methylation have been shown to play a vital role in tumorigenesis and disease progression in many malignancies, including TC (51, 52). Several studies have reported that specific genes exhibit differential methylation in TC, suggesting that these alterations may be useful in differentiating benign and malignant thyroid nodules (17, 53–55). Also, methylation status has been previously reported to affect the progression of thyroid cancer. Klein et al. have reported that distant metastatic differentiated thyroid cancer, poorly differentiated thyroid cancer, and anaplastic thyroid cancer (ATC) were increasingly affected by global hypomethylation, suggesting that this epigenetic entity may be involved in thyroid cancer progression and dedifferentiation (56). The most aggressive type of thyroid tumor, ATC, had been reported to show a global hypomethylation of the genome but with hypermethylation of CpG islands. Aberrant DNA methylation is common in ATC and likely contributes to tumorigenesis in this disease (57). Identification of clinically relevant cancer subtypes based on the DNA methylation pattern is of great significance in medicine, which may be helpful to provide specific and effective treatment options for patients with different subtypes. Here, in genome-wide DNA methylation analysis of 350 primary PTCs from TCGA database using the Illumina HumanMethylation450K platform, our study helped to identify two subtypes displayed markedly distinct DNA methylation levels, termed CIMP and nCIMP group. The CIMP tumors tend to have a higher degree of malignancy, since this subtype is tightly associated with older age, advanced pathological stage, and lymph node metastasis. Furthermore, differential methylation analysis showed a broad methylation gain in CIMP. Subsequent GGST analysis based on the significantly methylated probes in CIMP showed remarkable enrichment in epithelial mesenchymal transition and angiogenesis Hallmark pathways (**Supplementary Table S3**), confirming that the CIMP phenotype may promote the tumor progression from another perspective.

New discoveries in the field of tumor epigenetics have highlighted the critical role of DNA methylation in carcinogenesis, creating new opportunities to identify biomarkers for early cancer screening and personalized treatment. In recent years, many studies have shown that novel cancer biomarkers of DNA methylation could contribute to the early diagnosis and precise treatment of cancer, especially bowel and lung cancer (58). Meanwhile, there are increasing studies on the effect of increased methylation levels on the prognosis of PTC patients (59). Some studies have shown that demethylation agents are more promising in the treatment of aggressive thyroid cancer than traditional therapies (59). Researchers have made attempts in many aspects to achieve the clinical translation. For example, in clinical PTC biopsies, appropriate molecular markers can be found with the help of methylation microarray and bisulfite sequencing to detect early cancer or precancerous lesions and improve the understanding of tumorigenesis (60).

The CIMP phenotype was firstly described by Toyota et al. as an epigenetic phenotype in colorectal tumors characterized by significant hypermethylation in the promoter regions of tumor suppressor genes (18). Soon afterwards, the CIMP phenotype has been identified in many kinds of tumors, including glioma (20), renal cell carcinoma (21), gastric cancer (22), Pancreatic Cancer (23), and so forth. According to the researches, the CIMP tumor subset exhibits distinct clinicopathological, genomic/epigenomic, tumor-related immunity, and molecular features in relation to its nCIMP counterparts (19, 24, 61). Considering that CIMP may be a universal feature across different tumors, researchers applied a genome-wide unbiased and unsupervised hierarchical clustering of cancer-specific methylated CGI genes across 15 tumor types; however, PTC were not included in the cohort (62). To the best of our knowledge, this is the first study about the CIMP phenotype in PTC.

Recently, through the use of immunogenomics methods based on the transcriptomic and genomic data available in TCGA database, PTCs have been categorized as “inflammatory” tumors (63, 64). However, PTC are not tumors with high mutational burden, it is the substantial immune infiltrate that can account for the “inflammatory” immunoscore (64). A prominent feature of the CIMP subclass of PTC is a deficiency in antitumor immunity as evidenced by the significant inactivation of inflammatory response and interferon-γ Hallmark pathways (**Figure 2A**). Findings about the TME cell infiltration status also verified the view that CIMP subgroup was dramatically immune-depleted. All eight quantified infiltrating immune cells and two stromal cells were found to be significantly lower in CIMP than that of nCIMP (**Figure 2D**). Coincidentally, the TME has been implicated to play a critical role in cancer progression among several cancers (65, 66), including TC (67). Moreover, using independent external cohorts for validation, all three cohorts exhibited a “cold” TME phenotype that showed immune-depleted landscape (**Figures 4A–C**).

Accumulation of somatic mutations in oncogenes and tumor suppressor genes is common in the development and progression of cancer (68). Based on the mutation analysis, significantly higher somatic mutation burdens in specific genes were observed in patients of the CIMP subgroup, especially in NRAS, which have been shown to be major driver genes in PTC (44) (**Figure 5A**). The NRAS gene is the most frequent mutant gene of the RAS family and has been reported to be associated with an increased risk of distant metastasis (5). Additionally, CIMP had significantly higher TMB than that of nCIMP (**Figure 5B**). It is of great importance since that TMB is associated with immunotherapy response (69–71). TMB, defined as the total number of somatic coding errors, base substitutions, and indel mutations per million bases, can effectively estimate both overall mutational and neoantigen load (72, 73). Moreover, TMB can be used to predict immune checkpoint inhibitor therapy, acting as a biomarker of response to immunotherapy (74). Correspondingly, specific genes that represent potential targets for immunotherapy, such as PDCD1 (PD1), CD247 (CD3), CD274 (PDL1), were all at low expression level in CIMP (**Figure 2C**). As is known to all, current immunotherapy and particularly immune checkpoint blockers will be only effective for tumors with a pre-existing active immune response (75). In our study, we found CIMP presented with a lower enrichment level regarding immune and stromal cells (**Figures 2E, F**), as well as a lower fraction of tumor-infiltrating leukocytes (**Figure 2G**). These finds suggested that CIMP may not benefit from immunotherapy, as validated by the subclass mapping that the nCIMP showed high transcriptional similarity with a subgroup of melanoma patients who responded to anti-PD1 blockade. In contrast, there was no transcriptional similarity between CIMP PTCs and these melanoma patients (**Figure 3C**).

CNAs, refer to the gains and losses of DNA, are prevalent in cancer and may lead to chromosomal instability and aneuploidy (76, 77). These alterations have been implicated in cancer initiation, progression and therapeutic resistance (78, 79). In our study, we found the nCIMP subclass had significantly lower copy number loss or gain (**Figure 5C**), which indicated a better chromosomal stability. Consistently, analysis of focal level CNA landscape also supported the above views (**Figure 5D**). These findings, along with the discovery that CIMP had significantly higher TMB (**Figure 5B**), confirmed that there existed a close relationship between CIMP and genomic/epigenomic regulations.

The prognosis of PTC patients is still difficult to define, owing to the heterogeneity of the disease (80). The primary cause affecting the prognosis of PTC include age (81), tumor size (82, 83), extrathyroidal extension (84, 85), lymph node metastasis (86–88), distant metastasis (89–91), BRAF mutation (92, 93), TERT mutation (94, 95), and so on. Currently, there are no reliable biomarkers to accurately differentiate indolent thyroid tumors from more aggressive TCs. Therefore, identifying biomarkers for risk stratification of thyroid tumors may provide tools to reduce medical overtreatment and provide more effective therapies for the aggressive group. Researches have reported that CIMP may have prognostic significance (favourable or unfavourable) in terms of the OS/PFS or predictive value of therapeutic response in certain tumors (96–98). In our study, PTC patients with CIMP showed significantly poor clinical outcome as compared to nCIMP regarding OS (**Figure 1B**) and PFS (**Figure 1C**). Considering that PTC is a kind of well-differentiated malignancy with excellent prognosis, the significance of CIMP has been further highlighted. This finding, along with the results of COX regression analysis that CIMP remains an independent prognostic factor with respect to OS and PFS (**Figure 6**), rendering the CIMP a potential prognostic indicator for PTC.

## Conclusion

In summary, our data indicate that CIMP status stratify PTC patients into two distinct subgroups with distinct molecular and clinical phenotypes. The CIMP may modulate the immune response of the tumor microenvironment, influence the genomic heterogeneity and chromosomal instability, epigenetically silence the immune-related pathways, and thus affect the prognosis of PTC patients, which may help to make an assertion to provide specific and efficient treatment options for patients of different subtypes.

## Data availability statement

The raw data supporting the conclusions of this article will be made available by the authors, without undue reservation.

## Author contributions

PG and YZ were responsible for the analysis, interpretation of data, and graphing. PG and WZ drafted the manuscript. All authors read and approved the final manuscript. JC, XZ, and MG supervised the whole analysis and provided guidance and instructions. All authors contributed to the article and approved the submitted version.

## Funding

This work was supported by grants from the National Natural Science Foundation of China (81872169, 82172821, 82004158, 82103386), Tianjin Municipal Science and Technology Project (19JCYBJC27400, 21JCZDJC00360) and Beijing- Tianjin-Hebei Bas ic Research Cooperat ion Project (20JCZXJC00120), The Science & Technology Development Fund of Tianjin Education Commission for Higher Education (2021ZD033), Tianjin Medical Key Discipline (Specialty) Construction Project (TJYXZDXK-058B), and Tianjin Health Research Project (TJWJ2022XK024).

## Conflict of interest

The authors declare that the research was conducted in the absence of any commercial or financial relationships that could be construed as a potential conflict of interest.

## Publisher’s note

All claims expressed in this article are solely those of the authors and do not necessarily represent those of their affiliated organizations, or those of the publisher, the editors and the reviewers. Any product that may be evaluated in this article, or claim that may be made by its manufacturer, is not guaranteed or endorsed by the publisher.

## Supplementary material

Supplementary Figure 1 Bar plots showing the distribution between the CIMP/nCIMP phenotype and clinicopathological features: **(A)** pTNM stage; **(B)**pT stage and **(C)** pN stage.

Supplementary Figure 2 Scatter plot showing the generalized gene set testing (GGST) analysis between the the CIMP and nCIMP. The size of the dot represents the level of the differential expression (DE) of Hallmark pathways between the the CIMP and nCIMP.

## Abbreviations

CIMP, CpG island methylator phenotype; PTCs, papillary thyroid carcinomas; TC, thyroid carcinoma; CGIs, CpG islands; TCGA, the Cancer Genome Atlas; FPKM, fragments per kilobase million; OS, overall survival; PFS, progression-free survival; SD, standard deviation; MeTIL, methylation of tumor-infiltrating lymphocyte; FDR, false discovery rate; GGST, generalized gene set testing; GSEA, gene set enrichment analysis; CNA, copy number-altered; CNAs, copy number alterations; IC_50_, 50% inhibiting concentration; TME, tumor microenvironment; TMB, tumor mutation burden.

## References

[1] Global Burden of Disease Cancer, C Fitzmaurice C Abate D Abbasi N Abbastabar H Abd-Allah F. Global, regional, and national cancer incidence, mortality, years of life lost, years lived with disability, and disability-adjusted life-years for 29 cancer groups, 1990 to 2017: A systematic analysis for the global burden of disease study. JAMA Oncol (2019) 5:1749–68. doi: 10.1001/jamaoncol.2019.2996 PMC6777271 31560378

[2] Megwalu UC Moon PK. Thyroid cancer incidence and mortality trends in the united states: 2000-2018. Thyroid (2022) 32:560–70. doi: 10.1089/thy.2021.0662 35132899

[3] Ito Y Miyauchi A Kihara M Fukushima M Higashiyama T Miya A. Overall survival of papillary thyroid carcinoma patients: A single-institution long-term follow-up of 5897 patients. World J Surg (2018) 42:615–22. doi: 10.1007/s00268-018-4479-z PMC5801380 29349484

[4] Papp S Asa SL. When thyroid carcinoma goes bad: a morphological and molecular analysis. Head Neck Pathol (2015) 9:16–23. doi: 10.1007/s12105-015-0619-z 25804379 PMC4382495

[5] Xing M. Molecular pathogenesis and mechanisms of thyroid cancer. Nat Rev Cancer (2013) 13:184–99. doi: 10.1038/nrc3431 PMC3791171 23429735

[6] Van Nostrand D. Radioiodine refractory differentiated thyroid cancer: Time to update the classifications. Thyroid (2018) 28:1083–93. doi: 10.1089/thy.2018.0048 30105931

[7] Dralle H Machens A Basa J Fatourechi V Franceschi S Hay ID. Follicular cell-derived thyroid cancer. Nat Rev Dis Primers (2015) 1:15077. doi: 10.1038/nrdp.2015.77 27188261

[8] Esteller M. Epigenetics in cancer. N Engl J Med (2008) 358:1148–59. doi: 10.1056/NEJMra072067 18337604

[9] Rodriguez-Paredes M Esteller M. Cancer epigenetics reaches mainstream oncology. Nat Med (2011) 17:330–9. doi: 10.1038/nm.2305 21386836

[10] Timp W Bravo HC McDonald OG Goggins M Umbricht C Zeiger M. Large Hypomethylated blocks as a universal defining epigenetic alteration in human solid tumors. Genome Med (2014) 6:61. doi: 10.1186/s13073-014-0061-y 25191524 PMC4154522

[11] Gebhard C Mulet-Lazaro R Glatz D Schwarzfischer-Pfeilschifter L Schirmacher P Gaedcke J. Aberrant DNA methylation patterns in microsatellite stable human colorectal cancers define a new marker panel for the CpG island methylator phenotype. Int J Cancer (2022) 150:617–25. doi: 10.1002/ijc.33831 34591983

[12] Koch A Joosten SC Feng Z de Ruijter TC Draht MX Melotte V. Analysis of DNA methylation in cancer: location revisited. Nat Rev Clin Oncol (2018) 15:459–66. doi: 10.1038/s41571-018-0004-4 29666440

[13] Das PM Singal R. DNA Methylation and cancer. J Clin Oncol (2004) 22:4632–42. doi: 10.1200/JCO.2004.07.151 15542813

[14] Lee EK Chung KW Yang SK Park MJ Min HS Kim SW. DNA Methylation of MAPK signal-inhibiting genes in papillary thyroid carcinoma. Anticancer Res (2013) 33:4833–9. 24222120

[15] Chen J Liu C Yin L Zhang W. The tumor-promoting function of ECRG4 in papillary thyroid carcinoma and its related mechanism. Tumour Biol (2015) 36:1081–9. doi: 10.1007/s13277-014-2731-1 25326809

[16] Rodriguez-Rodero S Fernandez AF Fernandez-Morera JL Castro-Santos P Bayon GF Ferrero C. DNA Methylation signatures identify biologically distinct thyroid cancer subtypes. J Clin Endocrinol Metab (2013) 98:2811–21. doi: 10.1210/jc.2012-3566 23666970

[17] Cancer Genome Atlas Research, N Integrated genomic characterization of papillary thyroid carcinoma. Cell (2014) 159:676–90. doi: 10.1016/j.cell.2014.09.050 PMC4243044 25417114

[18] Toyota M Ahuja N Ohe-Toyota M Herman JG Baylin SB Issa JP. CpG island methylator phenotype in colorectal cancer. Proc Natl Acad Sci U.S.A. (1999) 96:8681–6. doi: 10.1073/pnas.96.15.8681 PMC17576 10411935

[19] Teodoridis JM Hardie C Brown R. CpG island methylator phenotype (CIMP) in cancer: causes and implications. Cancer Lett (2008) 268:177–86. doi: 10.1016/j.canlet.2008.03.022 18471961

[20] Noushmehr H Weisenberger DJ Diefes K Phillips HS Pujara K Berman BP. Identification of a CpG island methylator phenotype that defines a distinct subgroup of glioma. Cancer Cell (2010) 17:510–22. doi: 10.1016/j.ccr.2010.03.017 PMC2872684 20399149

[21] Linehan WM Ricketts CJ. The cancer genome atlas of renal cell carcinoma: findings and clinical implications. Nat Rev Urol (2019) 16:539–52. doi: 10.1038/s41585-019-0211-5 31278395

[22] Padmanabhan N Kyon HK Boot A Lim K Srivastava S Chen S. Highly recurrent CBS epimutations in gastric cancer CpG island methylator phenotypes and inflammation. Genome Biol (2021) 22:167. doi: 10.1186/s13059-021-02375-2 34074348 PMC8170989

[23] Ning G Li Y Chen W Tang W Shou D Luo Q. CpG island methylator phenotype modulates the immune response of the tumor microenvironment and influences the prognosis of pancreatic cancer patients. J Oncol (2021) 2021:2715694. doi: 10.1155/2021/2715694 34876903 PMC8645373

[24] Malta TM de Souza CF Sabedot TS Silva TC Mosella MS Kalkanis SN. Glioma CpG island methylator phenotype (G-CIMP): biological and clinical implications. Neuro Oncol (2018) 20:608–20. doi: 10.1093/neuonc/nox183 PMC5892155 29036500

[25] Zhang C Li Z Cheng Y Jia F Li R Wu M. CpG island methylator phenotype association with elevated serum alpha-fetoprotein level in hepatocellular carcinoma. Clin Cancer Res (2007) 13:944–52. doi: 10.1158/1078-0432.CCR-06-2268 17289889

[26] Wagner GP Kin K Lynch VJ. Measurement of mRNA abundance using RNA-seq data: RPKM measure is inconsistent among samples. Theory Biosci (2012) 131:281–5. doi: 10.1007/s12064-012-0162-3 22872506

[27] Sun J Shi R Zhang X Fang D Rauch J Lu S. Characterization of immune landscape in papillary thyroid cancer reveals distinct tumor immunogenicity and implications for immunotherapy. Oncoimmunology (2021) 10:e1964189. doi: 10.1080/2162402X.2021.1964189 34513318 PMC8425706

[28] Dom G Tarabichi M Unger K Thomas G Oczko-Wojciechowska M Bogdanova T. A gene expression signature distinguishes normal tissues of sporadic and radiation-induced papillary thyroid carcinomas. Br J Cancer (2012) 107:994–1000. doi: 10.1038/bjc.2012.302 22828612 PMC3464765

[29] Tarabichi M Saiselet M Tresallet C Hoang C Larsimont D Andry G. Revisiting the transcriptional analysis of primary tumours and associated nodal metastases with enhanced biological and statistical controls: application to thyroid cancer. Br J Cancer (2015) 112:1665–74. doi: 10.1038/bjc.2014.665 PMC4430711 25965298

[30] Giordano TJ Kuick R Thomas DG Misek DE Vinco M Sanders D. Molecular classification of papillary thyroid carcinoma: distinct BRAF, RAS, and RET/PTC mutation-specific gene expression profiles discovered by DNA microarray analysis. Oncogene (2005) 24:6646–56. doi: 10.1038/sj.onc.1208822 16007166

[31] Giordano TJ Au AY Kuick R Thomas DG Rhodes DR Wilhelm KG Jr. Delineation, functional validation, and bioinformatic evaluation of gene expression in thyroid follicular carcinomas with the PAX8-PPARG translocation. Clin Cancer Res (2006) 12:1983–93. doi: 10.1158/1078-0432.CCR-05-2039 16609007

[32] Zhou W Laird PW Shen H. Comprehensive characterization, annotation and innovative use of infinium DNA methylation BeadChip probes. Nucleic Acids Res (2017) 45:e22. doi: 10.1093/nar/gkw967 27924034 PMC5389466

[33] Tian Y Morris TJ Webster AP Yang Z Beck S Feber A. ChAMP: updated methylation analysis pipeline for illumina BeadChips. Bioinformatics (2017) 33:3982–4. doi: 10.1093/bioinformatics/btx513 PMC5860089 28961746

[34] Du P Zhang X Huang CC Jafari N Kibbe WA Hou L. Comparison of beta-value and m-value methods for quantifying methylation levels by microarray analysis. BMC Bioinf (2010) 11:587. doi: 10.1186/1471-2105-11-587 PMC3012676 21118553

[35] Yoshihara K Shahmoradgoli M Martinez E Vegesna R Kim H Torres-Garcia W. Inferring tumour purity and stromal and immune cell admixture from expression data. Nat Commun (2013) 4:2612. doi: 10.1038/ncomms3612 24113773 PMC3826632

[36] Jeschke J Bizet M Desmedt C Calonne E Dedeurwaerder S Garaud S. DNA Methylation-based immune response signature improves patient diagnosis in multiple cancers. J Clin Invest (2017) 127:3090–102. doi: 10.1172/JCI91095 PMC5531413 28714863

[37] Becht E Giraldo NA Lacroix L Buttard B Elarouci N Petitprez F. Estimating the population abundance of tissue-infiltrating immune and stromal cell populations using gene expression. Genome Biol (2016) 17:218. doi: 10.1186/s13059-016-1070-5 27765066 PMC5073889

[38] Phipson B Maksimovic J Oshlack A. missMethyl: an r package for analyzing data from illumina's HumanMethylation450 platform. Bioinformatics (2016) 32:286–8. doi: 10.1093/bioinformatics/btv560 26424855

[39] Liberzon A. The molecular signatures database (MSigDB) hallmark gene set collection. Cell Syst (2015) 1:417–25. doi: 10.1016/j.cels.2015.12.004 PMC4707969 26771021

[40] Ritchie ME. Limma powers differential expression analyses for RNA-sequencing and microarray studies. Nucleic Acids Res (2015) 43:e47. doi: 10.1093/nar/gkv007 25605792 PMC4402510

[41] Wu T. clusterProfiler 4.0: A universal enrichment tool for interpreting omics data. Innovation (Camb) (2021) 2:100141. doi: 10.1016/j.xinn.2021.100141 34557778 PMC8454663

[42] Xie Z. Gene set knowledge discovery with enrichr. Curr Protoc (2021) 1:e90. doi: 10.1002/cpz1.90 33780170 PMC8152575

[43] Lu X Meng J Zhou Y Jiang L Yan F. MOVICS: an r package for multi-omics integration and visualization in cancer subtyping. Bioinformatics (2020), btaa1018. doi: 10.1093/bioinformatics/btaa1018 33315104

[44] Bailey MH Tokheim C Porta-Pardo E Sengupta S Bertrand D Weerasinghe A. Comprehensive characterization of cancer driver genes and mutations. Cell (2018) 174:1034–5. doi: 10.1016/j.cell.2018.07.034 PMC8045146 30096302

[45] Mayakonda A Lin DC Assenov Y Plass C Koeffler HP. Maftools: efficient and comprehensive analysis of somatic variants in cancer. Genome Res (2018) 28:1747–56. doi: 10.1101/gr.239244.118 PMC6211645 30341162

[46] Mermel CH Schumacher SE Hill B Meyerson ML Beroukhim R Getz G. GISTIC2.0 facilitates sensitive and confident localization of the targets of focal somatic copy-number alteration in human cancers. Genome Biol (2011) 12:R41. doi: 10.1186/gb-2011-12-4-r41 21527027 PMC3218867

[47] Taylor AM Shih J Ha G Gao GF Zhang X Berger AC. Genomic and functional approaches to understanding cancer aneuploidy. Cancer Cell (2018) 33:676–689 e673. doi: 10.1016/j.ccell.2018.03.007 29622463 PMC6028190

[48] Silva TC Coetzee SG Gull N Yao L Hazelett DJ Noushmehr H. ELMER v.2: an R/Bioconductor package to reconstruct gene regulatory networks from DNA methylation and transcriptome profiles. Bioinformatics (2019) 35:1974–7. doi: 10.1093/bioinformatics/bty902 PMC6546131 30364927

[49] Roh W Chen PL Reuben A Spencer CN Prieto PA Miller JP. Integrated molecular analysis of tumor biopsies on sequential CTLA-4 and PD-1 blockade reveals markers of response and resistance. Sci Transl Med (2017) 379:eaah3560. doi: 10.1126/scitranslmed.aah3560 PMC5819607 28251903

[50] Geeleher P Cox NJ Huang RS. Clinical drug response can be predicted using baseline gene expression levels and *in vitro* drug sensitivity in cell lines. Genome Biol (2014) 15:R47. doi: 10.1186/gb-2014-15-3-r47 24580837 PMC4054092

[51] Klutstein M Nejman D Greenfield R Cedar H. DNA Methylation in cancer and aging. Cancer Res (2016) 76:3446–50. doi: 10.1158/0008-5472.CAN-15-3278 27256564

[52] Feinberg AP. The key role of epigenetics in human disease prevention and mitigation. N Engl J Med (2018) 378:1323–34. doi: 10.1056/NEJMra1402513 PMC11567374 29617578

[53] Beltrami CM Dos Reis MB Barros-Filho MC Marchi FA Kuasne H Pinto CAL. Integrated data analysis reveals potential drivers and pathways disrupted by DNA methylation in papillary thyroid carcinomas. Clin Epigenet (2017) 9:45. doi: 10.1186/s13148-017-0346-2 PMC5414166 28469731

[54] Bisarro Dos Reis M Barros-Filho MC Marchi FA Beltrami CM Kuasne H Pinto CAL. Prognostic classifier based on genome-wide DNA methylation profiling in well-differentiated thyroid tumors. J Clin Endocrinol Metab (2017) 102:4089–99. doi: 10.1210/jc.2017-00881 PMC5673278 28938489

[55] Buj R. Kallikreins stepwise scoring reveals three subtypes of papillary thyroid cancer with prognostic implications. Thyroid (2018) 28:601–12. doi: 10.1089/thy.2017.0501 29635968

[56] Klein Hesselink EN Mallona I Diez-Villanueva A Zafon C Mate JL Roca M. Increased global DNA hypomethylation in distant metastatic and dedifferentiated thyroid cancer. J Clin Endocrinol Metab (2018) 103:397–406. doi: 10.1210/jc.2017-01613 29165662

[57] Ravi N Yang M Mylona N Wennerberg J Paulsson K. Global RNA expression and DNA methylation patterns in primary anaplastic thyroid cancer. Cancers (Basel) (2020) 12:680. doi: 10.3390/cancers12030680 PMC7140095 32183222

[58] Gormally E Caboux E Vineis P Hainaut P. Circulating free DNA in plasma or serum as biomarker of carcinogenesis: practical aspects and biological significance. Mutat Res (2007) 635:105–17. doi: 10.1016/j.mrrev.2006.11.002 17257890

[59] Zhang K Li C Liu J Tang X Li Z. DNA Methylation alterations as therapeutic prospects in thyroid cancer. J Endocrinol Invest (2019) 42:363–70. doi: 10.1007/s40618-018-0922-0 29992502

[60] Zafon C Gil J Perez-Gonzalez B Jorda M. DNA Methylation in thyroid cancer. Endocr Relat Cancer (2019) 26:R415–39. doi: 10.1530/ERC-19-0093 31035251

[61] Issa JP. CpG island methylator phenotype in cancer. Nat Rev Cancer (2004) 4:988–93. doi: 10.1038/nrc1507 15573120

[62] Sanchez-Vega F Gotea V Margolin G Elnitski L. Pan-cancer stratification of solid human epithelial tumors and cancer cell lines reveals commonalities and tissue-specific features of the CpG island methylator phenotype. Epigenet Chromatin (2015) 8:14. doi: 10.1186/s13072-015-0007-7 PMC4424513 25960768

[63] Thorsson V Gibbs DL Brown SD Wolf D Bortone DS Ou Yang TH. The immune landscape of cancer. Immunity (2018) 48:812–830.e814. doi: 10.1016/j.immuni.2018.03.023 29628290 PMC5982584

[64] Liotti F Prevete N Vecchio G Melillo RM. Recent advances in understanding immune phenotypes of thyroid carcinomas: prognostication and emerging therapies. F1000Res (2019) 8:F1000 Faculty Rev-227. doi: 10.12688/f1000research.16677.1 PMC6396838 30854191

[65] Braun DA Street K Burke KP Cookmeyer DL Denize T Pedersen CB. Progressive immune dysfunction with advancing disease stage in renal cell carcinoma. Cancer Cell (2021) 39:632–648 e638. doi: 10.1016/j.ccell.2021.02.013 33711273 PMC8138872

[66] Hornburg M. Single-cell dissection of cellular components and interactions shaping the tumor immune phenotypes in ovarian cancer. Cancer Cell (2021) 39:928–944.e926. doi: 10.1016/j.ccell.2021.04.004 33961783

[67] Wu P Sun W Zhang H. An immune-related prognostic signature for thyroid carcinoma to predict survival and response to immune checkpoint inhibitors. Cancer Immunol Immunother (2022) 71:747–59. doi: 10.1007/s00262-021-03020-4 PMC10992838 34398303

[68] Vogelstein B Papadopoulos N Velculescu VE Zhou S Diaz LA Jr Kinzler K. Cancer genome landscapes. Science (2013) 339:1546–58. doi: 10.1126/science.1235122 PMC3749880 23539594

[69] Dong ZY Zhong WZ Zhang XC Su J Xie Z Liu SY. Potential predictive value of TP53 and KRAS mutation status for response to PD-1 blockade immunotherapy in lung adenocarcinoma. Clin Cancer Res (2017) 23:3012–24. doi: 10.1158/1078-0432.CCR-16-2554 28039262

[70] Rizvi NA Hellmann MD Snyder A Kvistborg P Makarov V Havel JJ. Cancer immunology. mutational landscape determines sensitivity to PD-1 blockade in non-small cell lung cancer. Science (2015) 348:124–8. doi: 10.1126/science.aaa1348 PMC4993154 25765070

[71] Snyder A Makarov V Merghoub T Yuan J Zaretsky JM Desrichard A. Genetic basis for clinical response to CTLA-4 blockade in melanoma. N Engl J Med (2014) 371:2189–99. doi: 10.1056/NEJMoa1406498 PMC4315319 25409260

[72] Zhang C Li Z Qi F Hu X Luo J. Exploration of the relationships between tumor mutation burden with immune infiltrates in clear cell renal cell carcinoma. Ann Transl Med (2019) 7:648. doi: 10.21037/atm.2019.10.84 31930049 PMC6944593

[73] Chan TA Yarchoan M Jaffee E Swanton C Quezada SA Stenzinger A. Development of tumor mutation burden as an immunotherapy biomarker: utility for the oncology clinic. Ann Oncol (2019) 30:44–56. doi: 10.1093/annonc/mdy495 30395155 PMC6336005

[74] Hugo W Zaretsky JM Sun L Song C Moreno BH Hu-Lieskovan S. Genomic and transcriptomic features of response to anti-PD-1 therapy in metastatic melanoma. Cell (2017) 168:542. doi: 10.1016/j.cell.2017.01.010 28129544

[75] Pardoll DM. The blockade of immune checkpoints in cancer immunotherapy. Nat Rev Cancer (2012) 12:252–64. doi: 10.1038/nrc3239 PMC4856023 22437870

[76] Sansregret L Swanton C. The role of aneuploidy in cancer evolution. Cold Spring Harb Perspect Med (2017) 7:a028373. doi: 10.1101/cshperspect.a028373 28049655 PMC5204330

[77] Levine MS Holland AJ. The impact of mitotic errors on cell proliferation and tumorigenesis. Genes Dev (2018) 32:620–38. doi: 10.1101/gad.314351.118 PMC6004076 29802124

[78] Davoli T Uno H Wooten EC Elledge SJ. Tumor aneuploidy correlates with markers of immune evasion and with reduced response to immunotherapy. Science (2017) 355:eaaf8399. doi: 10.1126/science.aaf8399 28104840 PMC5592794

[79] Beroukhim R Mermel CH Porter D Wei G Raychaudhuri S Donovan J. The landscape of somatic copy-number alteration across human cancers. Nature (2010) 463:899–905. doi: 10.1038/nature08822 20164920 PMC2826709

[80] Santoro M Melillo RM. Genetics: The genomic landscape of papillary thyroid carcinoma. Nat Rev Endocrinol (2015) 11:133–4. doi: 10.1038/nrendo.2014.209 25421371

[81] Adam MA. Exploring the relationship between patient age and cancer-specific survival in papillary thyroid cancer: Rethinking current staging systems. J Clin Oncol (2016) 34:4415–20. doi: 10.1200/JCO.2016.68.9372 PMC6366247 27998233

[82] Jeon MJ Thomas S Hyslop T Scheri RP Roman SA. Disease-specific mortality of differentiated thyroid cancer patients in Korea: A multicenter cohort study. Endocrinol Metab (Seoul) (2017) 32:434–41. doi: 10.3803/EnM.2017.32.4.434 PMC5744729 29199400

[83] Tran B Kim WG Kim TH Kim HK Kim BH Yi HS. The prognostic impact of tumor size in papillary thyroid carcinoma is modified by age. Thyroid (2018) 28:991–6. doi: 10.1089/thy.2017.0607 29921174

[84] Santos MJ Bugalho MJ. Papillary thyroid carcinoma: different clinical behavior among pT3 tumors. Endocrine (2016) 53:754–60. doi: 10.1007/s12020-016-0927-4 27000081

[85] Youngwirth LM Adam MA Scheri RP Roman SA Sosa JA. Extrathyroidal extension is associated with compromised survival in patients with thyroid cancer. Thyroid (2017) 27:626–31. doi: 10.1089/thy.2016.0132 27597378

[86] Adam MA Pura J Goffredo P Dinan MA Reed SD Scheri RP. Presence and number of lymph node metastases are associated with compromised survival for patients younger than age 45 years with papillary thyroid cancer. J Clin Oncol (2015) 33:2370–5. doi: 10.1200/JCO.2014.59.8391 26077238

[87] Lee J Song Y Soh EY. Prognostic significance of the number of metastatic lymph nodes to stratify the risk of recurrence. World J Surg (2014) 38:858–62. doi: 10.1007/s00268-013-2345-6 24305921

[88] Park YM Wang SG Lee JC Shin DH Kim IJ Son SM. Metastatic lymph node status in the central compartment of papillary thyroid carcinoma: A prognostic factor of locoregional recurrence. Head Neck (2016) 38 Suppl 1:E1172–1176. doi: 10.1002/hed.24186 26268535

[89] Durante C Haddy N Baudin E Leboulleux S Hartl D Travagli JP. Long-term outcome of 444 patients with distant metastases from papillary and follicular thyroid carcinoma: benefits and limits of radioiodine therapy. J Clin Endocrinol Metab (2006) 91:2892–9. doi: 10.1210/jc.2005-2838 16684830

[90] Maino F Forleo R Pacini F. Prognostic indicators for papillary thyroid carcinoma. Expert Rev Endocrinol Metab (2017) 12:101–8. doi: 10.1080/17446651.2017.1309278 30063426

[91] Wang LY Palmer FL Nixon IJ Thomas D Patel SG Shaha AR. Multi-organ distant metastases confer worse disease-specific survival in differentiated thyroid cancer. Thyroid (2014) 24:1594–9. doi: 10.1089/thy.2014.0173 25162180

[92] Huang Y Qu S Zhu G Wang F Liu R Shen X. BRAF V600E mutation-assisted risk stratification of solitary intrathyroidal papillary thyroid cancer for precision treatment. J Natl Cancer Inst (2018) 110:362–70. doi: 10.1093/jnci/djx227 PMC6658860 29165667

[93] Xing M Westra WH Tufano RP Cohen Y Rosenbaum E Rhoden KJ. BRAF mutation predicts a poorer clinical prognosis for papillary thyroid cancer. J Clin Endocrinol Metab (2005) 90:6373–9. doi: 10.1210/jc.2005-0987 16174717

[94] Liu R Xing M. TERT promoter mutations in thyroid cancer. Endocr Relat Cancer (2016) 23:R143–155. doi: 10.1530/ERC-15-0533 PMC4750651 26733501

[95] Moon S Song YS Kim YA Lim JA Cho SW Moon JH. Effects of coexistent BRAF(V600E) and TERT promoter mutations on poor clinical outcomes in papillary thyroid cancer: A meta-analysis. Thyroid (2017) 27:651–60. doi: 10.1089/thy.2016.0350 28181854

[96] Witte T Plass C Gerhauser C. Pan-cancer patterns of DNA methylation. Genome Med (2014) 6:66. doi: 10.1186/s13073-014-0066-6 25473433 PMC4254427

[97] Suzuki H Yamamoto E Maruyama R Niinuma T Kai M. Biological significance of the CpG island methylator phenotype. Biochem Biophys Res Commun (2014) 455:35–42. doi: 10.1016/j.bbrc.2014.07.007 25016183

[98] Miller BF Sanchez-Vega F Elnitski L. The emergence of pan-cancer CIMP and its elusive interpretation. Biomolecules (2016) 6. doi: 10.3390/biom6040045 PMC5197955 27879658

