# Supplementary figures and images for: Characterization of the CpG island methylator phenotype subclass in papillary thyroid carcinoma
Source: Front Endocrinol (Lausanne). 2022 Oct 24;13:1008301. doi: 10.3389/fendo.2022.1008301 (PMC9637834; doi:10.3389/fendo.2022.1008301)

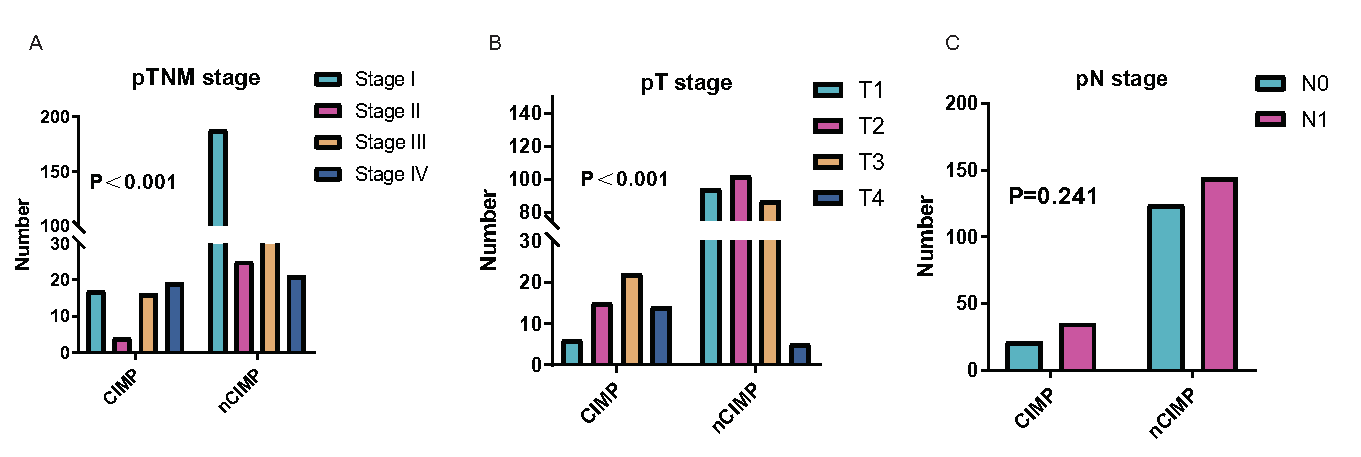

Supplement: Supplementary Figure 1 — Bar plots showing the distribution between the CIMP/nCIMP phenotype and clinicopathological features: (A) pTNM stage; (B)pT stage and (C) pN stage. [file Image_1.tiff]

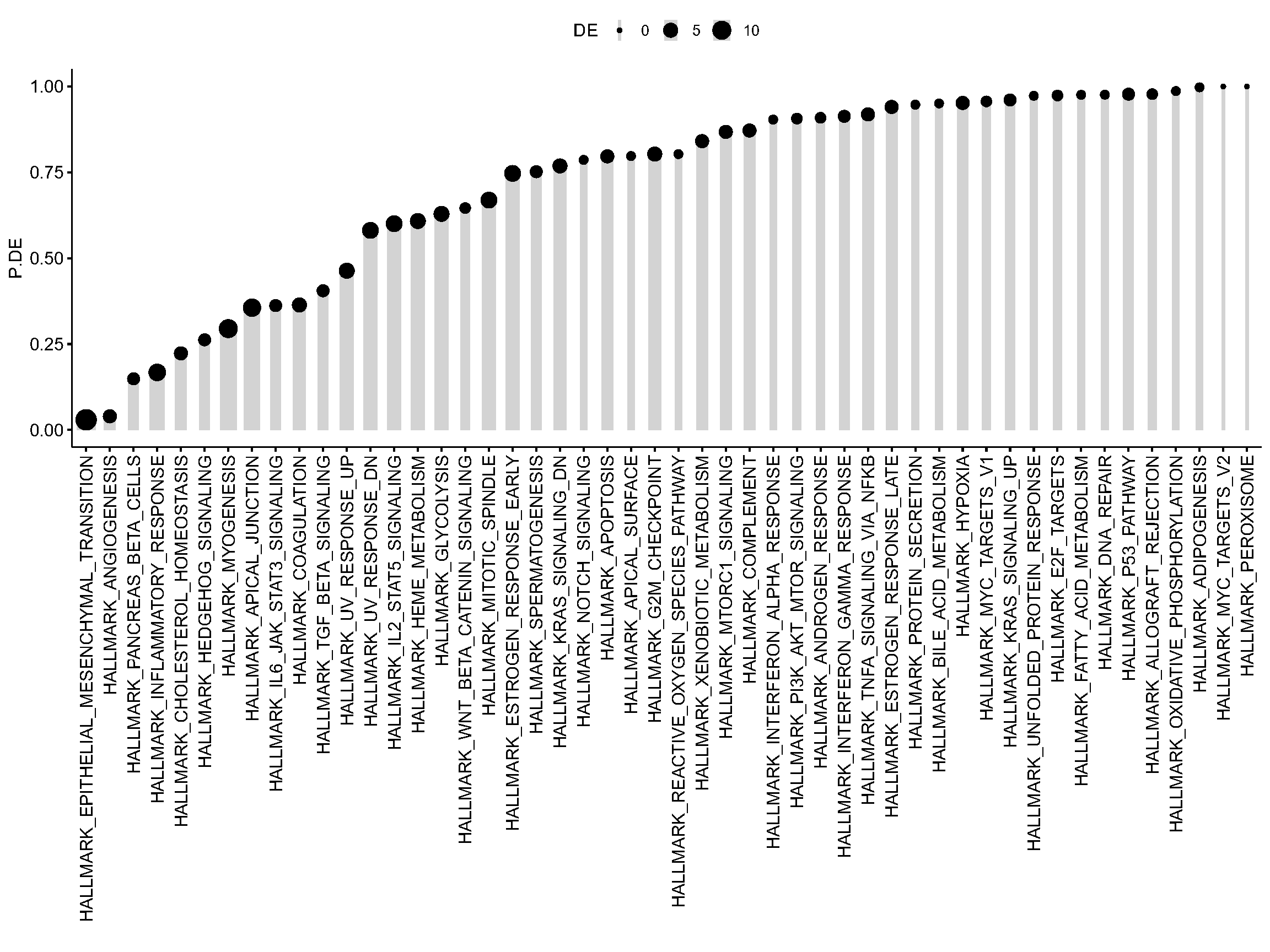

Supplement: Supplementary Figure 2 — Scatter plot showing the generalized gene set testing (GGST) analysis between the the CIMP and nCIMP. The size of the dot represents the level of the differential expression (DE) of Hallmark pathways between the the CIMP and nCIMP. [file Image_2.tiff]
